# Real-world efficacy and safety of nivolumab in previously-treated metastatic renal cell carcinoma, and association between immune-related adverse events and survival: the Italian expanded access program

**DOI:** 10.1186/s40425-019-0579-z

**Published:** 2019-04-03

**Authors:** Elena Verzoni, Giacomo Cartenì, Enrico Cortesi, Diana Giannarelli, Andrea De Giglio, Roberto Sabbatini, Sebastiano Buti, Sabrina Rossetti, Francesco Cognetti, Francesca Rastelli, Alberto Sobrero, Daniele Turci, Cora N. Sternberg, Camillo Porta, Federico Cappuzzo, Giampaolo Tortora, Davide Tassinari, Stefano Panni, Antonio Pazzola, Gianmarco Surico, Alessandra Raimondi, Ugo De Giorgi, Giuseppe Procopio, on behalf of the Italian Nivolumab Renal Cell Cancer Early Access Program group

**Affiliations:** 1 Medical Oncology-Genitourinary Unit, Fondazione IRCCS Istituto Nazionale dei Tumori di Milano, Via Venezian 1, 20133 Milan, Italy; 2 Oncology Unit, A. Cardarelli Hospital, Naples, Italy; 3 Radiology, Oncology and Pathology, Policlinico Umberto I, Rome, Italy; 4 Biostatistical Unit, Regina Elena National Cancer Institute – IRCCS, Rome, Italy; 5 Medical Oncology, Azienda Ospedaliera Santa Maria, Terni, Italy; 6 Oncology and Hematology Department, Azienda Ospedaliero-Universitaria Policlinico di Modena, Modena, Italy; 7 Medical Oncology, Azienda Ospedaliera di Parma, Parma, Italy; 8 Urology and Gynecology, Istituto Nazionale Tumori – IRCCS – Fondazione Pascale, Naples, Italy; 9 Medical Oncology, Regina Elena National Cancer Institute – IRCCS, Rome, Italy; 10 Medical Oncology, UOC Oncologia Area Vasta 4, Fermo, Italy; 11 Medical Oncology, San Martino Hospital, Genoa, Italy; 12 Medical Oncology, Ospedale Santa Maria delle Croci, Ravenna, Italy; 13 Medical Oncology, Azienda Ospedaliera S. Camillo Forlanini, Rome, Italy; 14 Department of Internal Medicine, University of Pavia and Division of Translational Oncology, IRCCS Istituti Clinici Scientifici Maugeri, Pavia, Italy; 15 Medical Oncology, Ospedale Umberto I (AUSL Romagna), Lugo, Italy; 16 Medical Oncology, AOU Integrata Verona “Borgo Roma”, Verona, Italy; 17 Oncology, Ospedale Infermi, Rimini, Italy; 18 Medical Oncology, Istituti Ospitalieri di Cremona, Cremona, Italy; 19 Medical Oncology, Ospedale Civile SS Annunziata, Sassari, Italy; 20 Medical Oncology, Ospedale Vito Fazzi, Lecce, Italy; 21 Urologic-Gynecologic Unit, Istituto Scientifico Romagnolo per lo Studio e la Cura dei Tumori (I.R.S.T.), Meldola, Italy

**Keywords:** Immunotherapy, Adverse events, Renal cell carcinoma, Expanded access trials

## Abstract

**Background:**

The Italian Renal Cell Cancer Early Access Program was an expanded access program that allowed access to nivolumab, for patients (pts) with metastatic renal cell carcinoma (mRCC) prior to regulatory approval.

**Methods:**

Pts with previously treated advanced or mRCC were eligible to receive nivolumab 3 mg/kg every 2 weeks. Pts included in the analysis had received ≥1 dose of nivolumab and were monitored for drug-related adverse events (drAEs) using CTCAE v.4.0. Immune-related (ir) AEs were defined as AEs displaying a certain, likely or possible correlation with immunotherapy (cutaneous, endocrine, hepatic, gastro-intestinal and pulmonary). The association between overall survival (OS) and irAEs was assessed, and associations between variables were evaluated with a logistic regression model.

**Results:**

A total of 389 pts were enrolled between July 2015 and April 2016. Overall, the objective response rate was 23.1%. At a median follow-up of 12 months, the median progression-free survival was 4.5 months (95% CI 3.7–6.2) and the 12-month overall survival rate was 63%. Any grade and grade 3–4 drAEs were reported in 124 (32%) and 27 (7%) of pts, respectively, and there were no treatment-related deaths. Any grade irAEs occurred in 76 (20%) of patients, 8% cutaneous, 4% endocrine, 2% hepatic, 5% gastro-intestinal and 1% pulmonary. Of the 22 drAEs inducing treatment discontinuation, 10 (45%) were irAEs. Pts with drAEs had a significantly longer survival than those without drAEs (median OS 22.5 versus 16.4 months, *p* = 0.01). Pts with irAEs versus without irAEs had a more significant survival benefit (median OS not reached versus 16.8 months, *p* = 0.002), confirmed at the landmark analysis at 6 weeks. The occurrence of irAEs displayed a strong association with OS in univariable (HR 0.48, *p* = 0.003) and multivariable (HR 0.57, *p* = 0.02) analysis.

**Conclusions:**

The appearance of irAEs strongly correlates with survival benefit in a real-life population of mRCC pts treated with nivolumab.

**Electronic supplementary material:**

The online version of this article (10.1186/s40425-019-0579-z) contains supplementary material, which is available to authorized users.

## Background

Nivolumab is a fully human monoclonal antibody targeting the programmed death receptor-1 (PD-1) and blocking the binding of PD-1, expressed on T cells, with its ligands PD-L1 and PD-L2, present on antigen-presenting cells and cancer cells [1]. Nivolumab therapy induces the disruption of PD-1/PD-L1 signaling, thus restoring the ability of T cells to selectively recognize and kill cancer cells [2].

Regarding metastatic renal cell carcinoma (mRCC), in a randomized phase III trial (CheckMate 025), nivolumab compared with everolimus proved able to confer a 5.4-month improvement of median OS, with a more favorable safety profile [3, 4]. The magnitude of the clinical benefit was so relevant that on November 2015 the Food and Drug Administration (FDA) and on April 2016 the European Medical Association (EMA) approved nivolumab for mRCC patients who had received a prior line of treatment with anti-angiogenic agents (Administration USFD, FDA Expands Use of Immunotherapeutic to Kidney Cancer) [5, 6]. Afterwards, the Italian Renal Cell Cancer Early Access Program (EAP) started in July 2015 based on such clinical data, during the evaluation of nivolumab by EMA and the negotiations with the Italian Ministry of Health [7].

Immune-checkpoint inhibitors (ICIs) induce a peculiar spectrum of toxicities, different from the one determined by conventional chemotherapy, caused by an enhanced activity of the immune system and by systemic inflammation: the so-called “immune-related adverse events” (irAEs) [8]. These adverse events (AEs) display a wide variety of manifestations concerning grade of toxicity, generally mild although severe cases may occur, as well as number and type of organs involved. IrAEs could be dermatological (rash, vitiligo and pruritus), gastrointestinal (diarrhea, colitis, hepatitis, increase of amylase and lipase), endocrine (thyroiditis and hypophysitis), pulmonary (pneumonitis), renal (nephritis) and systemic (fever and fatigue) [9].

In retrospective studies, non-conclusive evidence has been collected about a possible association of the occurrence of irAEs with durable responses and survival benefit from ICIs, both for anti-CTLA4 and anti-PD1, first in advanced melanoma [10–15] and then in other tumor settings, particularly non-small cell lung cancer (NSCLC) [16–18]. Recently, a prospective clinical trial demonstrated that, in NSCLC patients treated with nivolumab, the early occurrence of irAEs (specifically rash and pyrexia, but not diarrhea) correlated with an enhanced tumor response (37% vs 17%) and a longer progression-free survival (PFS) (6.4 vs 1.5 months). The authors reported a stronger predictive value for the detection of toxicities at two weeks from the treatment start, compared to the six-week assessment [19].

Given the controversy over the potential association of irAEs with favorable clinical outcomes and the discordant conclusions obtained in different cancer populations, here we report the results of a secondary analysis of the Italian EAP for nivolumab in mRCC patients, with the specific aim to assess the correlation of AEs, specifically irAEs, with patients’ outcome in this large real-life mRCC population.

## Patients and methods

### Study population

From July 2015 to April 2016 nivolumab was provided by BMS through the EAP in 95 hospitals in Italy. Totally, 490 requests were authorized, even though only 389 (80%) patients received at least one dose of nivolumab in this program [7].

Patients aged ≥18 years affected by advanced or mRCC that had relapsed after at least one prior therapy regimen (including, but not limited to, sunitinib, sorafenib, pazopanib, axitinib, tivozanib, bevacizumab) in the advanced or metastatic setting were considered eligible. Previous treatments with cytotoxics, mTOR inhibitors and cytokine therapy (e.g. IL-2, IFN), or vaccine therapy were also permitted. No limitation was given to the number of prior regimens. Any condition requiring systemic treatment with either corticosteroids (> 10 mg daily prednisone equivalent) or other immunosuppressive medications within 14 days prior to the first dose of study drug represented an exclusion criterion, while the presence of asymptomatic brain metastases or requiring systemic treatment with corticosteroids up to 10 mg daily prednisone equivalent within 14 days prior to the first dose of nivolumab was not. Mild impaired renal function was allowed including serum creatinine ≤1.5 x upper limit of normal or creatinine clearance ≥40 mL/min. Patients with active known or suspected autoimmune disease were excluded. All the patients included were requested to sign and date a written informed consent form provided by the company (BMS). The EAP received the approval by the Ethics Committee of each Center included in the program. All data presented here were prospectively collected on electronic patient files.

### Treatment modalities

Patients were administered treatment with nivolumab 3 mg/kg intravenously every 2 weeks until disease progression (PD), unacceptable toxicity, consent withdrawal, or physician’s decision based on clinical data.

Safety assessments were performed within 72 h prior to each nivolumab administration or as required by local standard of care procedures and included physical assessment and complete blood tests (hematology, renal and hepatic function, pancreatic enzymes and hormonal levels, specifically thyroid function testing including TSH reflex to free T3 and free T4 in case of abnormal result). Drug-related AEs (drAEs) were defined as all the AEs that the investigators classified as potentially related to treatment. Their incidence, grade and characteristics were obtained from patient clinical files and laboratory reports and classified according to the Common Terminology Criteria for AEs v4.0 (CTCAEs). Furtherly, the EAP investigators classified a subgroup of drAEs as irAEs, if they displayed a certain, likely or possible correlation with an immune-related pathogenesis, specifically considering five categories: cutaneous (rash/inflammatory dermatitis, bullous dermatosis, severe cutaneous adverse reactions, pruritus, vitiligo), endocrine (increased and/or decreased function of endocrine glands: thyroid, hypophysis and hypothalamic-pituitary axis, gonads, adrenal glands and pancreas), hepatic (hypertransaminasemia and hepatitis), gastro-intestinal (diarrhea, colitis, increased amylase and lipase) and pulmonary (pneumonitis, interstitial lung diseases, bronchiolitis obliterans organising pneumonia) toxicity.

### Endpoints

The primary endpoint of Italian EAP for nivolumab in mRCC was to assess the safety and efficacy of this agent in a real-world setting. The secondary endpoint was to assess the incidence of irAES in the real-life mRCC population treated with nivolumab, as well as the potential association of irAEs with patients’ outcome in terms of overall survival (OS).

### Statistical analysis

Data were summarized by frequency for categorical variables and by median and range for continuous variables. Continuous variables were compared using the Wilcoxon test. Association between categorical variables was assessed using the Fisher exact or the chi-square test, as appropriate. Differences were considered statistically significant when *p* < 0.05. PFS was calculated from the start of nivolumab until disease progression or death (whichever occurred first) or censored at the time of last follow-up. Patients discontinuing for toxicity were censored at the initiation of the subsequent therapy if still on response. OS was calculated from the start of nivolumab until death or censored at the time of last follow-up. Patients lost to follow-up were censored at the time of last contact. The Kaplan-Meier method was used to estimate PFS and OS. In order to minimize the bias related to drug exposure according to treatment duration, a landmark analysis at the median time appearance of AEs was performed. The log-rank test and Cox proportional hazards regression were used to test for differences between groups and to estimate hazard ratios and their 95% confidence intervals. Afterwards, univariable analysis a multivariable analysis was carried out by Cox regression model. When considering incidence of AEs, a logistic model was used to test associations with patients’ characteristics. In both regressions, only factors with a *P* value < 0.10 at the univariable analysis were included in the multivariable option. Multivariable analysis was implemented in a stepwise selection approach based on Wald statistics, with enter and remove *P* values set to 0.05 and 0.10, respectively. All statistical analyses were carried out with IBM-SPSS Statistical Software (IBM SPSS Statistics for Windows, Version 21.0. Armonk, NY).

## Results

A total of 389 patients were enrolled between July 2015 and April 2016 and treated with at least 1 dose of nivolumab, thus they represented the study population for this analysis. Baseline patients’ characteristics are reported in Table 1. The median follow-up was 11.9 months (range 1–24.7 months) and patients were administered a median of 13 doses of nivolumab (range 1–49 doses).

**Table 1:** Baseline patients characteristics

Characteristic	N (%)
Age	
< 75 years	319 (82.0)
≥ 75 years	70 (18.0)
Median (range) age, years 65 (34–85)	
Gender	
Male	291 (74.8)
Female	98 (25.2)
ECOG performance status	
0	176 (47.1)
1	174 (46.5)
2	24 (6.4)
NA	15
IMDC prognostic group	
Favourable	62 (20.2)
Intermediate	212 (69.1)
Poor	33 (10.7)
NA	82
Nephrectomy	
Yes	369 (94.9)
No	20 (5.1)
Histology	
Clear-cell	356 (91.5)
Non-clear-cell	26 (6.7)
Undifferentiated/Unknown	7 (1.8)
Metastasis site	
Lung	286 (73.5)
Lymph node	238 (69.2)
Bone	193 (49.6)
Liver	128 (32.9)
Brain	32 (8.2)
Number of prior systemic therapies	
1	80 (20.7)
2	137 (35.4)
≥ 3	170 (43.9)
First-line therapy	
Sunitinib	261 (67.4)
Pazopanib	80 (20.7)
Other	46 (11.9)
Prior everolimus	
Yes	163 (42.1)
No	224 (57.9)

Abbreviations: *N* number, *NA* not assessed, *ECOG* Eastern Cooperative Oncology Group, *IMDC* International Metastatic Renal Cell Carcinoma Database Consortium

Specific details regarding drAEs reported in the Italian EAP and in the Checkmate 025 trial are shown in Table 2. Any grade and grade 3–4 drAEs occurred in 124 (32%) and 27 (7%) of patients in Italian EAP, respectively. No treatment-related deaths were recorded. Median time to appearance of drAEs was 1.4 months (range 0–11.4 months) and they were generally manageable with treatment as per protocol-specific guidelines.

**Table 2:** Rates of drug-related adverse events reported in the CheckMate 025 trial and in the Italian Early Access Program of nivolumab in mRCC

	CheckMate025				Italian EAP	
	Everolimus *N* = 397		Nivolumab *N* = 406		Nivolumab *N* = 389	
	Any grade	Grade ≥ 3	Any grade	Grade ≥ 3	Any grade	Grade ≥ 3
Treatment-related AEs, %	88	37	79	19	32	7
Fatigue	34	3	33	2	13	2
Pyrexia	NR	NR	NR	NR	3	0
Nausea	17	1	14	< 1	0	0
Pruritus	10	0	14	0	0	0
Diarrhea	21	1	12	1	5	1
Decreased appetite	21	1	12	< 1	1	< 1
Rash	20	1	10	< 1	9	< 1
Hypothyroidism	NR	NR	NR	NR	2	0
Hyperthyroidism	NR	NR	NR	NR	2	0
Hypophisitis	NR	NR	NR	NR	< 1	< 1
Hypertransaminasemia	NR	NR	NR	NR	1	0
Cough	19	0	9	0	0	0
Anemia	24	8	8	2	2	< 1
Dyspnea	13	< 1	7	1	3	1
Edema peripheral	14	< 1	4	0	0	0
Pneumonitis	15	3	4	1	2	< 1
Mucosal inflammation	19	3	3	0	0	0
Dysgeusia	13	0	3	0	0	0
Hyperglycemia	12	3	2	1	< 1	< 1
Stomatitis	29	4	2	0	0	0
Hypertriglyceridemia	16	4	1	0	0	0
Epistaxis	10	0	1	0	0	0

Abbreviations: *NR* not reported, *N* number, *AEs* adverse events, *EAP* Early Access Program

DrAEs represented the reason for treatment discontinuation in 22 cases (7.9%), of which 10 (45%) were considered irAEs, including: grade 4 hyperglicemia (n = 1), grade 3 diarrhea (n = 1), grade 3 pneumonitis (n = 1), grade 3 bronchiolitis obliterans organising pneumonia (BOOP), grade 3 fatigue (n = 1), grade 3 skin toxicity (n = 1), grade 3 tremor (n = 1), grade 2 eyelid ptosis (n = 2), grade 2 liver toxicity (n = 1), grade 2 hypothyroidism (n = 1).

Regarding irAEs, any grade irAES occurred in 76 (20%) patients, of which 40 (10%) grade 1, 27 (7%) grade 2, 9 (2%) grade 3 and 1 (< 1%) grade 4 AEs, respectively. Considering the five pre-specified categories, 30 irAEs (8%) were cutaneous, 17 (4%) endocrine, 7 (2%) hepatic, 19 (5%) gastro-intestinal and 4 (1%) pulmonary. Further details are illustrated in Table 3.

**Table 3:** Rates of irAEs in the Italian Early Access Program of nivolumab in mRCC

irAEs	G1 N (%)	G2 N (%)	G3 N (%)	G4 N (%)	Any grade N (%)
Cutaneous	16 (4)	12 (3)	2 (1)	0	30 (8)
Endocrine	10 (3)	5 (1)	1 (< 1)	1 (< 1)	17 (4)
Hepatic	5 (1)	2 (1)	0	0	7 (2)
Gastro-intestinal	8 (2)	7 (2)	4 (1)	0	19 (5)
Pulmonary	1 (< 1)	1 (< 1)	2 (1)	0	4 (1)

Abbreviations: *N* number, *irAEs* immune-related adverse events

**Fig. 1:**
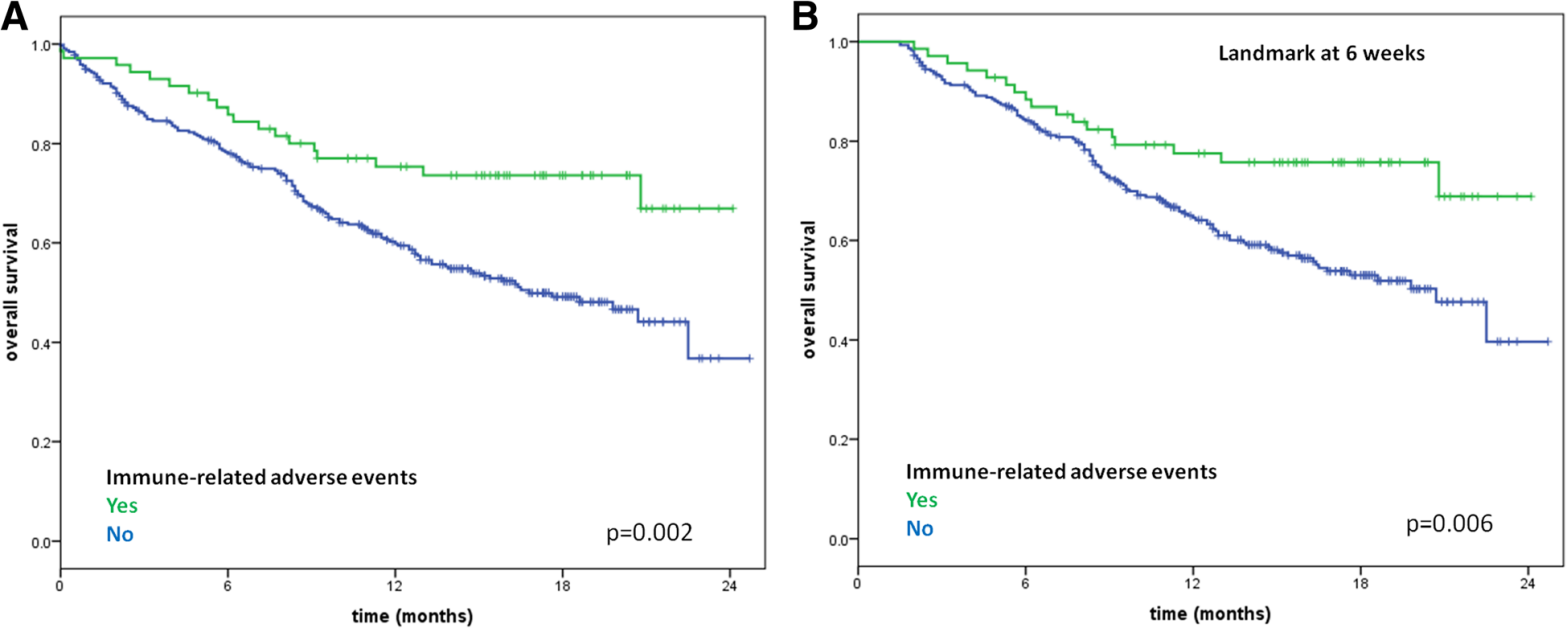
Survival analysis for immune-related adverse events. Kaplan-Meier curves for overall survival in patients stratified for the occurrence of immune-related adverse events (**a**) and with landmark analysis at 6 weeks (**b**)

At a median follow-up of 12 months, the median PFS was 4.5 months (95% CI 3.7–6.2 months) and the 1-year OS rate was 63% in the overall study population. In patients with and without drAEs, median OS was 22.5 months (95% CI not yet evaluable) versus 16.4 months (95% CI 12.1–20.7 months), 1-year OS was 69.0% versus 59.7% and 2-year OS was 46.6% versus 43.6%, respectively, *p* = 0.01 (Additional file 1: Figure S1A). In patients who discontinued treatment for the occurrence of drAEs, 1-year OS was 65%. A similar significant result was obtained considering selectively the population with the occurrence of irAEs (n = 76) versus no reported irAEs, where median OS was not reached versus 16.8 months (95% CI 13.0–20.6 months), 1-year OS was 75.4% versus 59.8% and 2-year OS was 66.9% versus 36.8%, respectively, *p* = 0.002 (Fig. 1a).

In order to minimize the bias related to drug exposure according to treatment duration, a landmark analysis at the median time appearance of AEs (6 weeks) was performed for OS. The appearance of drAEs showed a non-significant trend towards a prolonged OS (*p* = 0.71) (Additional file 1: Figure S1B) while irAEs retained their statistically significant association with improved OS (*p* = 0.006) (Fig. 1b).

In univariable model, a significant association between drAEs and prolonged OS was shown, with HR 0.64 (95% CI 0.46–0.91, *p* = 0.01) and, considering selectively patients experiencing irAEs, the correlation resulted to be stronger, with HR 0.48 (95% CI 0.30–0.78, *p* = 0.003). In a multivariable model, the significant association between irAEs and increased OS was confirmed, with HR 0.57 (95% CI 0.35–0.93, *p* = 0.02) (Table 4). A Cox regression analysis was used to estimate the association between survival and the available patients’ variables. Also, a variable related to the duration of treatment was considered in order to better understand the relationship between toxicity and OS, to confute the hypothesis that patients showing toxicity may be less or more treated than the others. Choosing 4 nivolumab doses as cut-off (corresponding to 6 weeks completed, the median time of appearance of AEs) the occurrence of a toxicity event maintains its prognostic role (Table 4).

**Table 4:** Cox model of association of baseline characteristics with overall survival

Characteristic	Univariate analysis		Multivariate analysis	
	HR (95% CI)	P value	HR (95% CI)	*P* value
Age, years				
≥ 75 vs < 75	0.62 (0.39–0.98)	0.04	0.55 (0.35–0.87)	0.01
Gender				
Male vs female	1.18 (0.82–1.71)	0.36	–	
Metastatic site, yes vs no				
Bone	1.35 (0.99–1.84)	0.06	–	
Liver	1.05 (0.76–1.46)	0.75	–	
CNS	1.39 (0.84–2.31)	0.20	–	
Number of prior therapies				
> 1 vs 1	1.80 (1.15–2.87)	0.01	–	
First line				
Sunitinib vs pazopanib	1.24 (0.84–1.84)	0.28		
Prior everolimus				
Yes vs no	1.30 (0.95–1.76)	0.10		
Drug-related toxicity (drAEs)				
Yes vs no	0.64 (0.46–0.91)	0.01	–	
Ir toxicity (irAEs)				
Yes vs no	0.48 (0.30–0.78)	0.003	0.57 (0.35–0.93)	0.02
Number of Nivolumab doses				
> 4 vs ≤4	0.11 (0.08–0.15)	< 0.0001	0.11 (0.08–0.15)	< 0.0001

Abbreviations: *CI* confidence interval, *HR* hazard ratio, *CNS* central nervous system

Considering the association of OS with the five pre-specified categories of irAEs, endocrine, cutaneous and gastrointestinal irAEs were associated with an improved OS (1-year OS 92.3, 81.6 and 78.6%, respectively), while hepatic and pulmonary irAEs conditioned a poor OS outcome (1-year OS 42.9 and 25.0%, respectively) (*p* = 0.001), confirmed at landmark analysis at 6 weeks, although the analysis was limited by the low numbers.

We analyzed the association with OS of drAEs and irAEs stratified for low grade (G1 and G2) and high grade (G3 and G4): 1-year OS in high versus low grade was 41.3% vs 75.5%, HR 2.44 (95% CI 1.26–4.72, *p* = 0.006) for drAEs, and 60.9% vs 79.6% HR 1.90 (95% CI 0.72–5.02, *p* = 0.19) for irAEs. Additionally, early onset (< 6 weeks) versus late onset (> 6 weeks) drAEs and irAEs showed 1-year OS rates of 70.7% vs 79.8% HR 1.96 (95% CI 0.87–4.43, *p* = 0.10) and 78.7% vs 85.2%, HR 1.76 (95% CI 0.54–5.74, *p* = 0.34), respectively.

## Discussion

We performed this analysis in order to assess the safety and efficacy of nivolumab in mRCC patients in the real-world setting in Italy, in an Early Access Program, and the potential association between irAEs and survival outcome.

Nivolumab proved to be safe and well-tolerated in the routine clinical practice. The most common drAEs occurred were fatigue, rash, diarrhea, thyroid disfunctions, both hypothyroidism and hyperthyroidism, and pyrexia, consistently with previous literature evidences regarding nivolumab treatment, including a recent pooled analysis on the safety profile of nivolumab in patients with advanced melanoma [15].

Interestingly, in our analysis we found a lower rate of drAEs in the Italian EAP, both any grade and grade ≥ 3, as compared to those of the nivolumab arm of the clinical trial Checkmate 025 [3, 4, 7]. These findings highlight the importance of real-world data since they are able to give key messages about the efficacy and safety results of new anti-cancer agents in the routine clinical practice, thus helping the development and the management of drugs.

We showed that patients reporting treatment-related toxicity had a significantly longer OS. The most remarkable finding is that the selection of AEs potentially determined by the nivolumab-related immune system activation, the irAEs, leads to observe a stronger association with an improved survival, confirmed in a multivariable model.

Several studies have reported a potential association between the occurrence of irAEs during immunotherapy and treatment efficacy. First, in the setting of advanced melanoma, the development of irAEs in patients treated with CTLA4 inhibitors proved to be significantly correlated with higher tumor response rate and probability of survival [10–13, 20]. Similarly, during treatment with nivolumab, patients experiencing any grade of irAEs displayed a significant overall survival (OS) improvement [14], whereas in a recent study any grade irAEs were associated with an increased overall response rate (ORR) but not with longer progression free survival (PFS) [15]. Afterwards, similar evidences were collected in non-small cell lung cancer (NSCLC) patients treated with nivolumab, where those experiencing irAEs had a higher ORR and an increased PFS, thus identifying the occurrence of irAEs as an independent predictor of treatment response [18]. Nevertheless, other evidences did not support the correlation between immune-related toxicity and benefit from immunotherapy. Specifically, in two cohorts of patients affected by metastatic melanoma treated with ipilimumab, the incidence of irAEs and the use of systemic corticosteroids to treat immune-related toxicity were not found to be associated with ORR, OS and time to treatment failure [21, 22].

Our findings support the association between irAEs and survival benefit from nivolumab. However, one of the possible bias of this analysis is the influence of treatment duration, since its increase leads to a prolonged nivolumab exposure and therefore to a potentially higher likelihood of AEs occurrence. To this purpose, we performed a landmark analysis at the median time of appearance of AEs, 6 weeks, and the association of irAEs occurrence with an improved OS was confirmed. Additionally, early versus late onset irAEs, defined as occurring before or after the cutoff of 6 weeks from the treatment start, showed a non-significant trend towards a poorer OS, however further studies should collect more solid evidence about this topic.

Interestingly, two recent studies obtained different results according to the intensity of the toxicity. Recently *Judd* et al*,* in a retrospective series of non-melanoma patients treated with anti-PD-1 agents obtained a trend towards an improved ORR in patients experiencing any irAE, but this association was statistically significant for low grade irAEs [23]. It confirmed the previous data by *Weber* et al, showing that in the setting of melanoma patients who received nivolumab monotherapy, those with any grade irAEs had a significantly better ORR, while for G3–4 irAEs this correlation was not significant [15]. This could reflect the tumor biolological aggressiveness, or, on the other side, it may be explained by the negative effects of high grade AEs, potentially dangerous or able to impair the adequate assessment of tumor response. In our results, G3–4 drAEs were significantly associated with a lower 1-year OS rate as compared to G1–2 drAEs, while G3–4 versus G1–2 irAEs showed a non-significant trend towards a shorter survival outcome.

The reasons underlying this potential association between irAEs occurrence and patients’ outcome are still to be determined, although some hypotheses have been advanced. IrAEs are caused by the unbalancing of the immune system induced by immune checkpoint blockade, possibly generated by the cross-reactivity between tumor neoantigens and normal tissue antigens [24]. These shared antigens could be involved in this process, although this seems not to be a convincing explanation in light of the low incidence rate of multiple irAEs in the same patient. Other possible mechanisms were postulated concerning anti-CTLA4 antibodies: on the one side the role of immunosuppressive regulatory T cells (T reg) is questioned, on the other ipilimumab could induce a non-specific increase in endogenous T-cell response mediated by dendritic cells or paracrine cytokine stimulation. Another interesting theory advocates a potential causality of this association irAEs-treatment efficacy due to the interaction between immunotherapy and polymorphisms in the genes involved in the response to ICIs [25]. Finally, considering anti-PD-1/PDL-1 inhibitors, they are deeply involved in the regulation of humoral immune response, since they proved able to modulate B cells both directly and via T-cell mediation. The altered production of auto-antibodies could in turn mediate the development of irAEs, thus possibly explaining the association between immune-related toxicity and treatment response [26]. A possible evidence in support of this hypothesis is the finding that the presence of pre-treatment anti-thyroid antibodies is an independent predictor of response to nivolumab in NSCLC patients [18].

Another key point is the definition of irAEs, since no well-established criteria have been identified yet and literature evidences are not clear upon this topic. We decided to select five distinct categories of irAEs (cutaneous, endocrine, hepatic, gastro-intestinal and pulmonary), according to the best known pathogenetic correlation between toxicity and immunotherapy. However, further studies focusing specifically on the mechanisms of action of ICIs could better clarify which AEs should be considered immune-related. The highest level of evidence collected in other disease settings regarding the association of irAEs with response to immunotherapy is about cutaneous toxicity, especially vitiligo, followed by endocrine alterations. Specifically, in melanoma patients, cutaneous irAEs were shown to be associated with clinical benefit and better outcome from anti-PD-1 treatment, with a major role of vitiligo [27–29] and in NSCLC dermatological and endocrine irAEs, specifically antibody-mediated thyroid disfunctions, resulted to be positively correlated with tumor responses or improved survival to anti-PD-1 ICIs [16, 17]. Although limited by the small numbers, we observed that cutaneous, endocrine and gastro-intestinal irAEs were significantly associated with improved OS, similarly to unselected irAEs, while hepatic and pulmonary irAEs conditioned a poor OS outcome. Nevertheless, no definitive conclusions could be derived from these results, considering the limited number of patients experiencing any specific class of irAEs.

At our knowledge, this is the first study investigating the potential association of irAEs with survival in the setting of mRCC. Our results highlight the importance to recognize treatment-related toxicities, specifically those potentially underlying immune-related mechanisms, since these display a significant association with an improved patients’ outcome from immunotherapy with anti-PD-1 ICIs. The limitations of this study are inherent to the specific nature of EAP, basically the high heterogeneity of the patients’ population and its retrospective nature, that could have impaired the power of the statistical analysis and limited to drive robust conclusions upon the results achieved. Moreover, as previously discussed, a precise definition and categorization of irAEs is still lacking, and the classification that we performed is not validated or standardized.

In conclusion, the appearance of irAEs strongly correlates with a survival benefit in a real-life population of mRCC patients treated with nivolumab. Further studies could confirm the potential role of the incidence of irAEs as a predictor of response to ICIs as well as explore the underlying mechanisms leading to the development of immune-related toxicity. As a consequence, criteria should be identified to establish whether a precise timing of occurrence of irAEs or specific organ-related toxicities could better predict the treatment outcome, with the aim to better personalize the treatment management in this disease setting.

## Additional file

Additional file 1: **Figure S1.** Kaplan-Meier curves for overall survival in patients stratified for the occurrence of drug-related adverse events (A) and with landmark at 6 weeks (B). (TIF 343 kb)

## Abbreviations

AEs: Adverse events
CI: Confidence interval
CTCAEs: Common Terminology Criteria for AEs
drAEs: Drug-related AEs
EAP: Early Access Program
EMA: European Medical Association
FDA: Food and Drug Administration
HR: Hazard ratio
ICIs: Immune-checkpoint inhibitors
irAEs: Immune-related adverse events
mRCC: Metastatic renal cell carcinoma
NSCLC: Non-small cell lung cancer
ORR: Overall response rate
OS: Overall survival
PD: Disease progression
PD-1: Programmed death receptor-1
PD-L1: Programmed death-ligand 1
PFS: Progression free survival
Treg: Regulatory T cells
VEGF: Vascular endothelial growth factor
