# Supplementary figures and images for: Real-world efficacy and safety of nivolumab in previously-treated metastatic renal cell carcinoma, and association between immune-related adverse events and survival: the Italian expanded access program
Source: J Immunother Cancer. 2019 Apr 3;7:99. doi: 10.1186/s40425-019-0579-z (PMC6448290; doi:10.1186/s40425-019-0579-z)

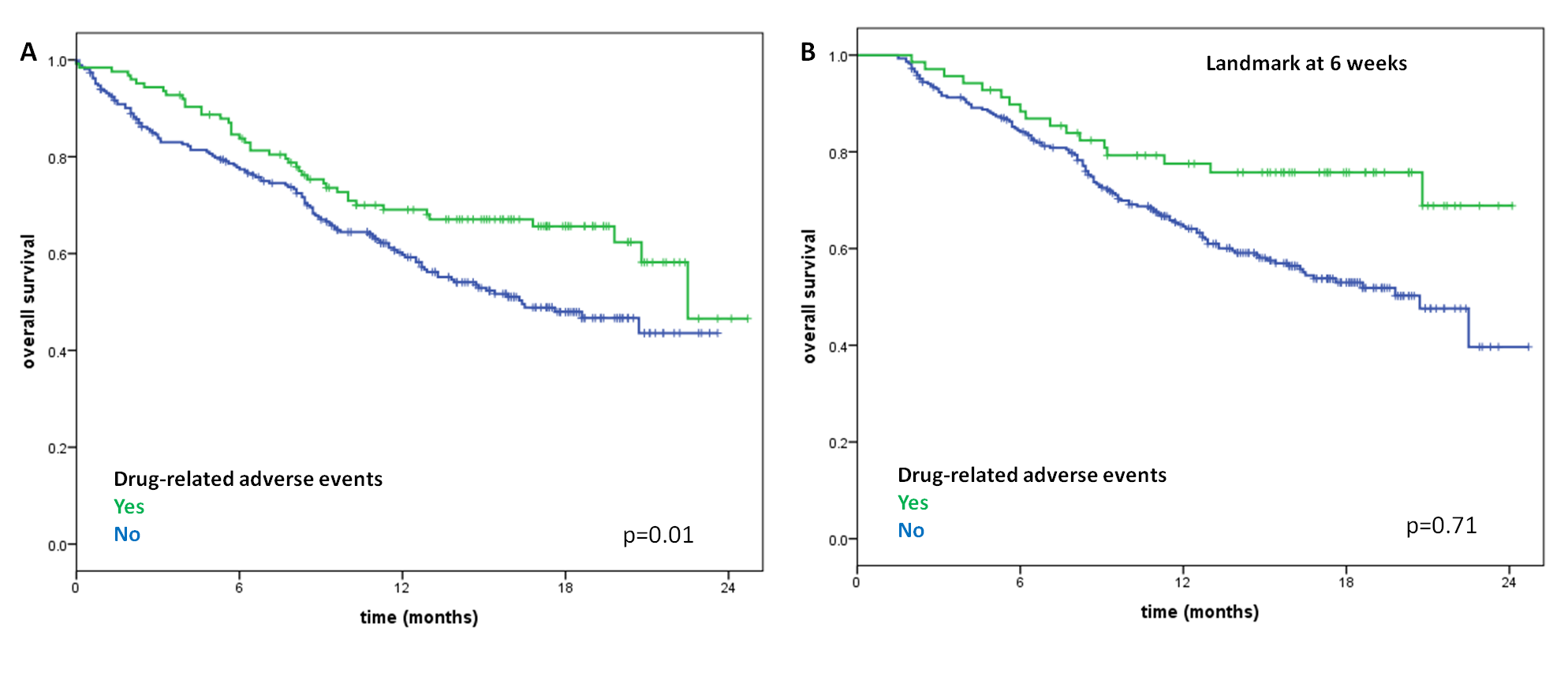

Supplement: Supplementary file 1 — Figure S1. Kaplan-Meier curves for overall survival in patients stratified for the occurrence of drug-related adverse events (A) and with landmark at 6 weeks (B). (TIF 343 kb) [file 40425_2019_579_MOESM1_ESM.tif]
